# Combating COVID-19 Vaccine Hesitancy: A Synthetic Public Segmentation Approach for Predicting Vaccine Acceptance

**DOI:** 10.1017/dmp.2022.282

**Published:** 2022-12-21

**Authors:** Myoung-Gi Chon, Sungsu Kim

**Affiliations:** 1 School of Communication & Journalism, Auburn University, Auburn, Alabama, USA; 2 School of Communication, Kookmin University, Seoul, South Korea

**Keywords:** COVID-19 vaccination, lacuna public, public segmentation, institutional trust, fear

## Abstract

**Objective:**

Vaccine hesitancy impacts the ability to cope with coronavirus disease 2019 (COVID-19) effectively in the United States. It is important for health organizations to increase vaccine acceptance. Addressing this issue, this study aimed to predict citizens’ acceptance of the COVID-19 vaccine through a synthetic approach of public segmentation including cross-situational and situational variables. Controlling for demographics, we examined institutional trust, negative attitudes toward, and low levels of knowledge about vaccines (ie, lacuna public characteristics), and fear of COVID-19 during the pandemic. Our study provides a useful framework for public segmentation and contributes to risk and health campaigns by identifying significant predictors of COVID-19 vaccine acceptance.

**Method:**

We conducted an online survey on October 10, 2020 (*N* = 499), and performed hierarchical regression analyses to predict citizens’ COVID-19 vaccine acceptance.

**Results:**

This study demonstrated that trust in the Centers for Disease Control and Prevention (CDC) and federal government, vaccine attitude, problem recognition, constraint recognition, involvement recognition, and fear positively predicted COVID-19 vaccine acceptance.

**Conclusions:**

This study outlines a useful synthetic public segmentation framework and extends the concept of lacuna public to the pandemic context, helping to predict vaccine acceptance. Importantly, the findings could be useful in designing health campaign messages.

Coronavirus disease 2019 (COVID-19) vaccination is the most effective solution in the continuing pandemic; thus, increasing vaccination rates is a pressing issue.^1^ Despite the importance of vaccination for controlling and ending the pandemic, numerous individuals in the United States are still reluctant to get vaccinated.^2^ Naturally, it is critical for health authorities to promote vaccination against COVID-19, which can be achieved by identifying significant factors predicting vaccination decisions and delineating the characteristics of vaccine acceptors and rejectors.^3^ This public segmentation approach helps public health organizations to identify target groups (ie, key public segments) and develop effective tailored intervention strategies enhancing willingness to obtain COVID-19 vaccines.^4,5^

In strategic communication research, public segmentation has been widely used to identify target groups.^6^ In health campaigns, communication practitioners in health organizations aim to segment a group of people who are exposed to a given health problem.^7^ For example, public segmentation has been used to design messages to improve cancer prevention.^8^ For government crisis communication, public segmentation has also been used to predict individuals’ advocatory or adversarial megaphoning behaviors toward the government (ie, positive or negative word-of-mouth about the government).^4^ Given the background of public segmentation, we suggest that it can be applied in health risk communication to predict COVID-19 vaccine acceptance.

Thus, this study proposes a synthetic segmentation framework to predict vaccination intentions, which is key to combating vaccine hesitancy in the context of an infectious disease outbreak. The synthetic public segmentation framework entails 2 general approaches: cross-situational and situational approaches.^9^ Cross-situational segmentation uses the factors whose changes are not based on how individuals perceive themselves in a given situation (eg, socio-demographics, psychographics, knowledge), whereas situational segmentation relies on someone’s characteristics that depend specifically on situational perceptions about themselves (eg, problem recognition, involvement recognition).^4^ Indeed, as a recommended public segmentation method, the synthesis of these 2 approaches boosts each one’s strengths.^9^ More importantly, it is necessary to reflect on the crisis context to predict outcomes.^4^ Even though situational factors pertaining to crisis-specific issues are regarded as more useful than cross-situational ones in general situations, segmentation by the former only may not maximize interventions’ effectiveness.^9^ In this regard, when health practitioners aim to increase COVID-19 vaccination rates, they should understand what makes individuals reluctant to obtain vaccines during the COVID-19 pandemic particularly. The synthetic application should include individual characteristics—related to infectious diseases and vaccines as cross-situational or situational factors—to predict vaccine acceptance or hesitancy precisely.

## Cross-Situational Factors in Public Segmentation

Along with socio-demographics, this study examines institutional trust (ie, trust in the Centers for Disease Control and Prevention [CDC] and federal government) and vaccine-related knowledge and attitudes as cross-situational factors of public segmentation. Trust in health authorities and government is significant in enhancing crisis management, because infectious disease mitigation depends on the public’s compliance with specified guidelines.^10^ Governmental trust indicates a positive public-organization relationship, which has been found to predict compliance with recommended behaviors during the pandemic.^10^ More germane to this study, recent literature has demonstrated that greater trust in the government is positively associated with COVID-19 vaccine acceptance (ie, intentions to receive the vaccine).^11,12^ Considering that the federal government (including the CDC) is at the forefront of nationwide efforts to manage the COVID-19 pandemic, this study focuses on public trust in the CDC and federal government as an indicator of vaccine acceptance. We hypothesized that CDC trust (H1) and federal government trust (H2) would be associated with COVID-19 vaccination intentions.

Additionally, this study applies the concept of lacuna public, which refers to a subgroup of activists with negative attitudes and deficient knowledge about an issue.^13^ Drawing on the notion of lacuna individuals, we used knowledge deficiency, vaccine negativity, and issue activeness to predict individuals’ communication behaviors (eg, information seeking, sharing, and forwarding) with respect to vaccine issues.^14^ However, little attention has been paid to the influences of the lacuna public’s characteristics on vaccination decision-making. Thus, this study first applies the cross-situational indicators of the lacuna public to COVID-19 vaccine acceptance. We posed the following research question: Are levels of knowledge about general vaccines and attitudes toward the COVID-19 vaccine positively associated with COVID-19 vaccination intentions?

## Situational Factors in Public Segmentation

Along with knowledge about and attitudes toward COVID-19 vaccines, individuals’ activeness is another critical factor to explain the lacuna public.^14^ More specifically, 3 perception-based situational variables encompass how individuals are active or engaged with a particular issue: perception of the phenomenon as a problem (ie, problem recognition), perception of the obstacles to solving the problem (ie, constraint recognition), and perception of their connection with the issue (ie, involvement recognition).^4^ Research has shown these variables to affect communication and compliance behaviors with the government,^10,14^ but there has been a relative lack of research investigating the effects in the context of COVID-19 vaccination decision-making. Thus, we posed an additional research question: Are problem recognition, constraint recognition, and involvement recognition positively associated with COVID-19 vaccination intentions?

We also suggest fear of COVID-19 as a critical predictor of COVID-19 vaccine acceptance, which falls under our synthetic public segmentation approach. Despite the importance of negative emotions which have been used to predict public behaviors, little research has investigated the role of negative emotions in the public segmentation framework. In particular, scholars in risk and health communication have demonstrated that fear, guilt, and disgust are significant determinants of health behaviors.^15^ Particularly, fear has been identified as a major determinant of engagement in recommended health behaviors in the context of infectious disease outbreak, as a prediction of future situations can evoke feelings of fear.^10^ Applying a risk-based theoretical framework to the COVID-19 pandemic, research has demonstrated that those with greater fear are more likely to have stronger intentions to receive the vaccine.^16,17^ As fear of COVID-19 is universally high in the United States,^18^ it likely predicts vaccine acceptance. Hence, we hypothesized that fear of COVID-19 infection would be positively associated with COVID-19 vaccination intentions (H3).

## Methods

We conducted an online survey on October 10, 2020, when vaccines were not released and still undergoing development in the United States^19^ This study was approved by the Institutional Review Boards for the Protection of Human Subjects in Research (IRB) of Auburn University (Protocol #: 20-285 EX 2006). Upon the IRB approval, we recruited US participants and administered the survey by means of Amazon Mechanical Turk (MTurk). All participants provided informed consent before starting the survey. The total sample size was 499 (63.7% male). The mean age of the sample was 35.66 y (*SD* = 10.25; range = 18-74). Among the respondents, 46.3% (*n* = 231) reported an annual household income of $50,000 or below, 48.1% (*n* = 240) reported an income between $50,001 and $90,000, and 5.6% (*n* = 28) reported an income above $90,000. The median education level was a 4-y degree. Approximately 52.3% (*n* = 261) reported identifying as Republicans, 38.3% (*n* = 191) as Democrats, and 9.4% (*n* = 47) as Independents.

### Measures

We averaged responses to create an index for each of the following variables unless otherwise indicated (see Table 1 for measurement items).

**Table 1.** Measurement items for key variables

Variable	Measurement items
Institutional trust (CDC and federal government)	- This organization treats people like me fairly and justly. - Whenever this organization makes an important decision, I know it will be concerned about people like me. - This organization can be relied on to keep its promises. - I believe this organization takes the opinions of people like me into account when making decisions. - This organization has the ability to accomplish what it says it will do.
Vaccine knowledge	- Vaccines are superfluous, as diseases can be treated (eg, with antibiotics). - Without broadly applied vaccine programs, smallpox would still exist. - The efficacy of vaccines has been proven. - The doses of the chemicals used in vaccines are not dangerous for humans. - Vaccinations increase the occurrence of allergies.
Vaccine attitudes	- Bad-Good - Unimportant-Important - Harmful-Beneficial - Foolish-Wise - Threatening-Assuring - Risky-Safe
Problem recognition	- This issue is a serious social and national problem. - This issue should be dealt with more seriously by the government and related organizations. - There should be immediate efforts to resolve this issue. - I am very concerned about this issue.
Constraint recognition	- The government and related organizations will consider opinions from persons like me on this issue. - If I try, opinions from person like me on this issue can affect regulations related to this issue. - I (my efforts) can help in resolving this issue. - I feel that my ideas or opinions matter to those who are working on this issue.
Involvement recognition	- This issue (Vaccination) is significantly related to me. - This issue potentially affects my family members/friends. - I am connected with this problem and its consequences. - I think this issue could affect me personally.
Fear of COVID-19 infection	- I am afraid of COVID-19 infection. - I am frightened by COVID-19 infection. - I am scared of COVID-19 infection.
COVID-19 vaccination intentions	- How likely is it that you will try to get more information about the COVID-19 vaccine? - How likely is it that you will consider getting the COVID-19 vaccine? - How likely is it that you will make it a priority to get the COVID-19 vaccine? - How likely is it that you will actually get the COVID-19 vaccine, once available? - What is the likelihood you will get the COVID-19 vaccine if a health care provider offers it to you within 3 y?

### Cross-Situational Factors

Socio-demographic measures included gender (1 = male; 0 = female), age, income, education level, and party identification (1 = Republican; 0 = non-Republican).

To measure institutional trust, we used 2 sets of 5 items each to assess trust in the CDC and the federal government, respectively.^20^ For both CDC trust (Cronbach’s α = .89; *M* = 3.92; *SD* = 0.69) and federal government trust (Cronbach’s α = .86; *M* = 3.89; *SD* = 0.74), participants responded on a scale ranging from 1 (“strongly disagree”) to 5 (“strongly agree”).

As a vaccine-specific factor, we assessed general vaccine knowledge levels with 5 items.^21^ Knowledge about specific COVID-19 vaccines was not captured, because the survey had taken place before COVID-19 vaccines were actually available.^19^ We summed the number of correct answers (*M* = 2.66; *SD* = 1.13) to create the index of the vaccine knowledge. As another vaccine-specific factor, attitudes toward receiving the COVID-19 vaccine were measured by using 6 items on a 5-point semantic differential scale (bad-good, unimportant-important, harmful-beneficial, foolish-wise, threatening-assuring, and risky-safe; Cronbach’s α = .82; *M* = 4.06; *SD* = 0.69) were used.^22,23^

### Situational Factors

We measured 3 activeness indicators about the given issue, COVID-19 vaccination, on a 7-point Likert scale ranging from 1 (“not at all”) to 5 (“very much”).^10,14,24,25^ We used sets of 4 items each to assess problem recognition (Cronbach’s α = .64; *M* = 3.93; *SD* = 0.63), constraint recognition (Cronbach’s α = .77; *M* = 3.77; *SD* = 0.77), and involvement recognition (Cronbach’s α = .72; *M* = 3.83; *SD* = 0.70), respectively.

As another situational factor, fear of COVID-19 infection was measured by using 3 items.^26^ Participants responded to each item on a 5-point Likert scale ranging from 1 (“strongly disagree”) to 5 (“strongly agree”) (Cronbach’s α = .74; *M* = 3.79; *SD* = 0.81).

### COVID-19 Vaccination Intentions

To measure COVID-19 vaccination intentions, we used 5 items adapted from an existing scale. Responses were captured on a 5-point Likert scale ranging from 1 (“very unlikely”) to 5 (“very likely”).^27,28^

## Results

Table 2 presents the correlations among the main study variables. Then, to examine individual characteristics’ prediction of behavioral intentions to accept COVID-19 vaccines, we performed hierarchical regression analyses (see Table 3). Among the socio-demographics, age was significantly associated with COVID-19 vaccination intentions (β = .11; *P* < 0.05). When entering socio-demographics as covariates, CDC trust (β = .47; *P* < 0.001) and federal government trust (β = .24; *P* < 0.001) were significantly and positively related to COVID-19 vaccination intentions, supporting H1 and H2. The results of the analyses testing the effects of cross-situational factors of the lacuna public showed that general vaccine knowledge did not significantly influence vaccination intentions (β = .01; *P* > 0.05), but attitudes toward COVID-19 vaccines were a significant positive predictor of vaccination intentions (β = .45; *P* < 0.001), after controlling for socio-demographics and institutional trust. Additionally, controlling for the effects of socio-demographics, institutional trust, and vaccine-specific factors, there were significant positive influences of problem recognition (β = .25; *P* < 0.001) and involvement recognition (β = .16; *P* < 0.001) and a negative influence of constraint recognition (β = –.12; *P* < 0.01) on vaccination intentions. Fear of COVID-19 infection (β = .26; *P* < 0.001) was a significant positive driver of vaccination intentions, after controlling for all other variables entered in previous blocks. Hence, H3 was supported.

**Table 2.** Zero-order correlations among study variables (*N* = 499)

Variables	1	2	3	4	5	6	7	8	9
1. CDC trust	1.00								
2. Federal government trust	.54***	1.00							
3. Vaccine knowledge	.16***	–.11*	1.00						
4. Vaccine attitudes	.51***	.36***	.26***	1.00					
5. Problem recognition	.56***	.33***	.22***	.54***	1.00				
6. Constraint recognition	.53***	.70***	–.11*	.40***	.52***	1.00			
7. Involvement recognition	.53***	.37***	.14**	.45***	.68***	.52***	1.00		
8. Fear of COVID-19	.46***	.35***	.07	.43***	.47***	.37***	.50***	1.00	
9. Vaccination intentions	.60***	.48***	.15**	.64***	.62***	.45***	.57***	.61***	1.00

*P < 0.05.

**P < 0.01.

***P < 0.001.

**Table 3.** Hierarchical regression analyses predicting COVID-19 vaccination intentions (*N* = 499)

	COVID-19 vaccination intentions				
	Model 1	Model 2	Model 3	Model 4	Model 5
Block 1: Socio-demographics^a^					
Gender (Male = 1)	–.03	–.02	–.02	–.01	–.02
Age	.11*	.04	.05	.03	.01
Income	–.01	.01	.03	.01	.04
Education	.07	–.07	–.10**	–.10**	–.10**
Party affiliation (Republican = 1)	.05	.02	.01	.01	.03
Inc. *R*^2^ (%)	2.2				
Block 2: Institutional trust^a^					
CDC trust		.47***	.27***	.15***	.12**
Federal government trust		.24***	.20***	.24***	.21***
Inc. *R*^2^ (%)		37.7***			
Block 3: Vaccine-specific factors^a^					
Vaccine knowledge			.01	–.03	–.01
Vaccine attitudes			.45***	.35***	.31***
Inc. *R*^2^ (%)			14.6***		
Block 4: Activeness indicators^b^					
Problem recognition				.25***	.21***
Constraint recognition				–.12**	–.11*
Involvement recognition				.16***	.10*
Inc. *R*^2^ (%)				6.5***	
Block 5: Negative emotion^b^					
Fear of COVID-19					.26***
Inc. *R*^2^ (%)					4.1***
Total *R*^2^ (%)					65.0

*Note*. Cell entries for all models are standardized regression coefficients. ^a^indicates cross-situational factors and ^b^ indicates situational factors.

*P < 0.05.

**P < 0.01.

***P < 0.001.

## Discussion

This study aimed to develop an integrated framework of public segmentation to predict individuals’ acceptance of COVID-19 vaccines by using a synthetic approach including cross-situational and situational variables. We found that CDC trust, federal government trust, attitudes, problem recognition, constraint recognition, involvement recognition, and fear of COVID-19 were positively related to individuals’ acceptance of COVID-19 vaccines. The results have several theoretical and practical implications.

Our findings confirm the validity of the synthetic framework of public segmentation in the context of the COVID-19 pandemic. Cross-situational and situational factors were both significant in predicting COVID-19 vaccination intentions, providing additional empirical evidence for the segmentation methods recommended in prior literature.^4,9^ The synthetic approach’s theoretical and empirical efforts are necessary to reflect static and dynamic environmental factors surrounding a given issue.^9^ The findings suggest that individuals are more likely to follow health authorities’ vaccination recommendations when their cross-situational (eg, high trust in CDC) and situational (eg, high recognition of involvement with COVID-19) characteristics are simultaneously activated and motivate specific decisions.

Notably, including institutional trust as a variable contributes to the extension of the public segmentation approach. Trust in organizations is forged over the history of the organization-public relationship.^20^ Studies have paid little attention to institutional trust in predicting vaccination behaviors during a public health crisis. This study provides empirical evidence of the effect of trust in the federal government on the acceptance of COVID-19 vaccines and implies that the government may leverage its trustworthiness when devising targeted health interventions. Indeed, those who trust the federal government are more likely to follow governmental instructions about COVID-19. Citizens with good relationships with the CDC are more likely to follow infectious disease mitigation guidelines.^10^ As trust is 1 factor explaining the organization-public relationship’s quality in strategic communication,^20^ our findings demonstrate why governments should secure citizens’ trust before a public health crisis occurs. An existing trust may act as an available resource for health authorities to utilize when they are implementing interventions for public health crises.

Another noteworthy contribution is that the findings shed light on how various characteristics of the lacuna public differentially predict individuals’ COVID-19 vaccine acceptance. Knowledge levels, attitudes, and activeness are characteristics comprising the lacuna public and used in predicting communicative behaviors on vaccine issues.^14^ To our knowledge, this study was the first attempt to apply the concept in predicting vaccination intentions. Our findings provide insights for applying the concept of lacuna public to vaccine acceptance, focusing on attitudes and activeness. In terms of health interventions to increase COVID-19 vaccination rates, strategically reaching out to those with negative attitudes toward and low issue activeness regarding vaccination would be an efficient approach.

Of interest, the effect of general vaccine knowledge on COVID-19 vaccination intentions was not significant. Individuals’ knowledge deficiency has been identified as a critical element in conceptualizing a group of vocal individuals (ie, the lacuna public),^13,14^ but the present findings suggest that it may not be applicable in predicting vaccination intentions. In this regard, as the first study to examine how lacuna individuals’ characteristics relate to vaccine acceptance, this study’s results suggest that individuals’ vaccine attitudes could be a stronger determinant of vaccine acceptance than issue-related knowledge, but further verification is needed.

It is also notable that this study examined the public’s fear of COVID-19 infection, extending the existing segmentation framework to predict vaccine acceptance during the COVID-19 pandemic. The conventional public segmentation approach highlights cognitive situational factors, while emotional aspects garner the least attention, even though fear of contracting disease has been found to influence health behaviors.^29^ As a discrete emotion widely studied in health and risk communication, we found fear of contracting COVID-19 to be a significant predictor of vaccination intentions, which aligns with previous findings.^17^ One study found that, during a hypothetical infectious disease outbreak in the United States, individuals were motivated to receive and transmit health information, following CDC instructions.^10^ Extending prior studies’ identification of fear’s role in health and risk communication, the current study suggests that fear plays a vital role in predicting individuals’ vaccine acceptance during the ongoing pandemic.

**Figure 1.**
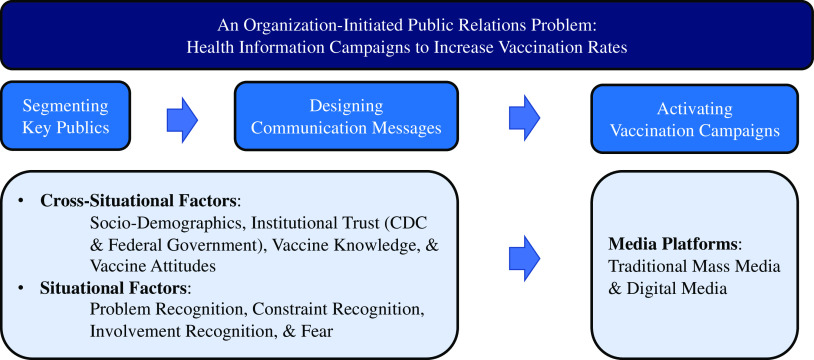
A guideline of strategic segmentation to predict vaccine acceptance.

Practically, this study’s results can guide risk communicators in the public sector in understanding the public’s attitudes and behaviors and designing campaign messages promoting COVID-19 vaccine acceptance. It is important to focus on the characteristics of individuals who want to accept COVID-19 vaccines, as indicated by their attitudes toward and engagement with the vaccine issue during the COVID-19 pandemic. Particularly, 3 situational variables are derived from the situational theory of problem solving.^25^ The theory suggests that individuals who are vocal about a particular issue emerge and engage in active communicative behaviors when they recognize the existence of a problem (problem recognition), perceive fewer barriers to addressing the problem (constraint recognition), and perceive their connectedness to the problem (involvement recognition).^25^ As these situational variables can be used to segment the public according to varying levels of issue activeness,^14^ our findings indicate that they are essential for identifying those who will accept or reject COVID-19 vaccines. Importantly, fear of COVID-19 and trust in health authorities should also be considered significant characteristics of COVID-19 vaccine acceptors. Furthermore, specific factors of the segmentation framework may inform public health interventions during the pandemic. Identifying target segments’ characteristics and tailoring campaign messages accordingly would enhance the effectiveness of interventions to influence individuals’ health behaviors.^8^ In the context of this study, for example, messages intentionally delivered from non-government sources (eg, private medical practitioners) may be effective for individuals who are highly engaged with the COVID-19 issue but do not trust the federal government (Figure 1).

To use the proposed synthetic model of public segmentation effectively, public health communicators need to identify key publics and design communication messages before activating their health information campaigns. According to scholars, a health information campaign is regarded as an organization-initiated public relations problem recognized by an organization.^7^ Based on its recognition of the problem affecting people, the public health organization likely aims to increase the COVID-19 vaccination rates.^7^ Accordingly, the synthetic model of public segmentation can be used to segment key publics (ie, target publics) and design campaign messages during an infectious disease crisis. Figure 1 gives practical insights and guidance to take advantage of the synthetic model of public segmentation during public health campaigns. Overall, this study’s results contribute to the development of a synthetic framework of public segmentation to predict COVID-19 vaccine acceptance. Practically, this study also suggests the potential utility of tailored messages to increase COVID-19 vaccine rates.
